# The Moisture in the Air

**Published:** 1863-10

**Authors:** 


The Moisture in the Air.—One of the most curious and interesting of the recent discoveries of science is, that it is to the presence of a very small portion of watery vapor in our atmosphere—less than one-half of one per cent.—that much of the beneficient effect of the heat is due. The rays of heat sent forth from the earth after it has been warmed by the sun, would soon be lost in space, but for the wonder ful absorbent properties of these molecules of aqueous vapor, which act with many thousand times the power of the atoms of oxygen and nitrogen, of which the air is composed. By this means, the heat instead of being transmitted into infinitude as fast as produced, is stopped, or dammed up, and held back on its course, to furnish the necessary conditions of life and growth. Let this moisture be taken from the air but for a single summer night, and the sun would rise the next morning upon a "world fast in the grip of frost." But the power of absorption and of radiation in the same body are always equal, so that at length it is poured forth into space, else our atmosphere would become a vast reservoir of fire, and all organic life be burned up.—*Med. and Surg. Rep.*

